# *Pinus pinaster* Early Hormonal Defence Responses to Pinewood Nematode (*Bursaphelenchus xylophilus*) Infection

**DOI:** 10.3390/metabo11040227

**Published:** 2021-04-08

**Authors:** Ana M. Rodrigues, Swen Langer, Isabel Carrasquinho, Ed Bergström, Tony Larson, Jane Thomas-Oates, Carla António

**Affiliations:** 1 Plant Metabolomics Laboratory, Instituto de Tecnologia Química e Biológica António Xavier, Universidade Nova de Lisboa (ITQB NOVA), Av. da República, 2780-157 Oeiras, Portugal; amrodrigues@itqb.unl.pt; 2 Centre of Excellence in Mass Spectrometry, University of York, Heslington, York YO10 5DD, UK; swen.langer@york.ac.uk (S.L.); ed.bergstrom@york.ac.uk (E.B.); tony.larson@york.ac.uk (T.L.); jane.thomas-oates@york.ac.uk (J.T.-O.); 3 Department of Biology, Bioscience Technology Facility, University of York, Heslington, York YO10 5DD, UK; 4 Instituto Nacional de Investigação Agrária e Veterinária, I. P. (INIAV), Edifício Estação Florestal Nacional, Av. da República, Quinta do Marquês, 2780-159 Oeiras, Portugal; isabel.carrasquinho@iniav.pt; 5 Linking Landscape, Environment, Agriculture and Food (LEAF), Instituto Superior de Agronomia (ISA), Universidade de Lisboa (ULisboa), Tapada da Ajuda, 1349-017 Lisboa, Portugal; 6 Department of Chemistry, University of York, Heslington, York YO10 5DD, UK; 7 Centre for Novel Agricultural Products, Department of Biology, University of York, Heslington, York YO10 5DD, UK

**Keywords:** pine wilt disease, maritime pine, biotic stress, plant metabolomics, forest tree metabolomics, phytohormones, mass spectrometry (MS), quantitative MS, analytical method validation, triple quadrupole

## Abstract

The pinewood nematode (PWN) is the causal agent of pine wilt disease, a pathology that affects conifer forests, mainly *Pinus* spp. PWN infection can induce the expression of phytohormone-related genes; however, changes at the early phytohormone level have not yet been explored. Phytohormones are low-abundance metabolites, and thus, difficult to quantify. Moreover, most methodologies focus mainly on Arabidopsis or crop species. This work aimed to validate a fast (run time 6.6 min) liquid chromatography-triple quadrupole tandem mass spectrometry (LC-QqQ-MS/MS) analytical method to quantify 14 phytohormones in *Pinus pinaster* stem tissues. This method was further applied to evaluate, for the first time, early phytohormone changes in susceptible and resistant phenotypes of *P. pinaster* 24, 48 and 72 h after inoculation (HAI) with PWN. A significant increase in salicylic acid (SA, 48 and 72 HAI) and jasmonic acid methyl ester (JA-ME, 72 HAI) was observed in susceptible phenotypes. Results indicate that the higher susceptibility of *P. pinaster* to PWN infection might result from an inefficient trigger of hypersensitive responses, with the involvement of JA and SA pathways. This work provides an important update in forest research, and adds to the current knowledge of *Pinus* spp. defence responses to PWN infection.

## 1. Introduction

The pine wilt disease (PWD) is a devasting pathology caused by the pinewood nematode (PWN) *Bursaphelenchus xylophilus* (Steiner and Buhrer) Nickle, that affects conifer forests, mainly *Pinus* spp., with severe ecological and economic impact. Once inside the tree, nematodes multiply rapidly, invade the resin canals, and lead to the complete collapse of the vascular system in just a few weeks [1]. After the spread of the PWN from its native area (North America) to the Far East, and to Portugal in 1999 [2], several preventative measures have been imposed to avoid the dispersion to European forests; namely, wood trade regulations, buffer zones around affected areas, and cutting down of symptomatic and dead trees [3,4,5]. Although variation in resistance to PWN infection between and within European *Pinus* spp. has been documented [6,7,8,9,10], little is known about the biochemical mechanisms underlying differential tree susceptibility.

Secondary metabolites (e.g., terpenes, phenolic compounds) are well-known for their role in environmental responses; namely, biotic stress and plant defence regulation [11]. The analysis of terpenes in the essential oil of *Pinus* spp. in response to PWN infection revealed no major differences between control and inoculated pine hosts [8,12]. However, other secondary metabolites, such as phenolic acids, condensed tannins and lignin, have been associated with tree resistance and constitutive chemical defences [13,14,15,16]. A putative role of phytohormones in PWD defence mechanisms has also been proposed [13]. Moreover, transcriptomic studies showed that PWN infection induced the expression of phytohormone-related genes in susceptible *Pinus* spp. [17,18,19,20]. However, there have been no further advances at the hormonal level to understanding how the levels of these defence-related metabolites change after PWN infection.

Phytohormones are low-abundance secondary metabolites with critical signalling roles in response to internal and external cues; namely, in mediating plant growth and development, adaptation to adverse environmental conditions, and defence responses [21,22]. The major phytohormone classes include auxins (AXs), gibberellins (GAs), cytokinins (CKs), abscisic acid (ABA), ethylene (ET), salicylic acid (SA), jasmonic acid (JA), brassinosteroids (BRs) and strigolactones. In general, AXs, GAs, ET, CKs and BRs are responsible for crucial plant growth and developmental processes; SA, JA and ET are known to play major roles in regulating plant defence responses to biotic stresses, while ABA is mostly responsible for plant responses to environmental stresses (e.g., drought, salinity, wounding) [21,22,23]. Additionally, signalling interactions/crosstalk among phytohormones are known to occur in the control of numerous growth, developmental and defence processes [24,25,26,27].

Phytohormones have diverse chemical structures and are present at very low concentrations in plants (pg/g to ng/g fresh weight range), which makes them challenging metabolites to quantify accurately [28,29]. Moreover, most published analytical methods for quantitative analysis of phytohormones are validated mainly for Arabidopsis or crop species [30,31] using plant leaf material that often contains higher analyte concentrations than other tissues (e.g., stems, roots) [28,32]. Thus, improvements in sensitive analytical techniques able to quantify several phytohormones in a single run and in different plant species and tissues, such as those from forest trees, are of utmost importance.

Liquid chromatography coupled to tandem mass spectrometry (LC-MS/MS) has become the method of choice for the targeted analysis and quantification of most phytohormones [31,33,34,35], with the triple quadrupole tandem MS system (QqQ-MS/MS) often employed to discriminate multiple phytohormones with different precursor and product ion *m*/*z* values [34]. The QqQ-MS technology enables highly sensitive detection using selected reaction monitoring (SRM) experiments, by observing specific precursor-to-product ion transitions for each phytohormone, and similar transitions for the respective internal standards (IS), at a high sampling rate for accurate quantification.

To date, only three analytical methods for the quantification of phytohormones have been validated for complex matrices such as those from forest tree species [36,37,38]. However, they lack either extensive phytohormone coverage or simplicity of the extraction protocol. In addition, technical difficulties have been reported during phytohormone extraction when using forest tree tissues [38]. The complexity of a phytohormone quantitative analysis is mainly due to the numerous structurally similar compounds and/or potentially interfering compounds present in the plant matrix [38]. Thus, the development of MS-based methods for the analysis of multiple phytohormones has gained increasing interest. The methodology described here provides a simple extraction protocol coupled to a fast analytical method (run time 6.6 min) using an LC-QqQ-MS/MS system for the quantitative analysis of 14 phytohormones from six major classes in the complex stem matrix of 1-year-old maritime pine (*Pinus pinaster* Ait.) half-sib plants, and that can be easily applied to large-scale studies (e.g., breeding programs). The phytohormones included in this study were (i) ABA, (ii) SA and its immediate precursor benzoic acid (BA), (iii) gibberellic acid (GA) and gibberellin A_9_ (GA_9_) from the GA biosynthetic pathway, (iv) indole-3-acetic acid (IAA), its precursor indole-3-butyric acid (IBA) and the respective ester conjugates indole-3-acetic acid methyl ester (IAA-ME) and indole-3-butyric acid methyl ester (IBA-ME), (v) JA and its conjugated form jasmonic acid methyl ester (JA-ME), (vi) *trans*-zeatin (Zea), its precursor *N*_6_-isopentenyladenine (iP), and the conjugated *trans*-zeatin riboside (ZeaR). The method was further applied to study maritime pine early hormonal defence responses to PWN infection using non-inoculated, mock-inoculated and PWN-inoculated 2-year-old *P. pinaster* half-sib plants, to assess the role of these metabolites in PWD progression.

This work provides an important update to the analytical methodologies currently being used in forest research, and supports the hypothesis that *P. pinaster* susceptibility to PWN infection might result from an inefficient activation of hypersensitive responses (HR), with the involvement of the JA and SA pathways.

## 2. Results

### 2.1. LC-QqQ-MS/MS Analytical Method Validation

In this study, a LC-QqQ-MS/MS method was developed and validated for the quantification of 14 phytohormones (from six major classes) in *P. pinaster* stem tissues, namely ABA, BA, GA, GA_9_, IAA, IAA-ME, IBA, IBA-ME, JA, JA-ME, iP, SA, Zea, and ZeaR. Phytohormones were extracted using methanol: H_2_O (70:30, *v*/*v*) and analysed directly without further purification. The ability of the developed LC-QqQ-MS/MS method to quantify low-abundance phytohormones in the complex matrix of *P. pinaster* stem tissues was evaluated following standard key analytical method validation steps; namely determination of (i) linearity, (ii) matrix effects, (iii) limit of detection (LOD) and limit of quantification (LOQ), (iv) analytical recoveries, and (v) method precision [39]. In this study, ionisation efficiency was determined for each analyte, collision energy settings were optimised, and the two most intense SRM transitions obtained for each analyte were chosen as the quantification transition and confirmation transition (Table 1). Resulting SRM chromatograms of the 14 target phytohormones in a standard mixture solution are presented in Supplementary Material Figure S1.

#### 2.1.1. Linearity

The linearity of the analytical method (extraction and response) was established by performing calibration with internal standardisation (in solvent and in *P. pinaster* stem matrix). The range of calibration values used was defined individually for each analyte, based on the amount of each compound present in the plant matrix. Overall, the method showed good linearity over the concentration range (in solvent and in matrix extract), with determination coefficients (*R*^2^) higher than 0.990 in solvent and in matrix (Table 2). Residual plots were also used to evaluate linearity, where the residual of each point in the calibration curve (i.e., the difference between the calculated and theoretical values) is plotted against the respective concentration level (Supplementary Figures S2 and S3). In a linear model, the residuals of the regression are expected to be normally distributed and any curvature suggests a lack of fit due to a non-linear effect [40]. In general, residual plots did not show particular patterns, with residual values tending to cluster towards the middle of the plot, and close to 0 on the Y-axis, therefore no weighting factor was applied. This indicates that the measurement error is the same and normally distributed for each sample (homoscedasticity).

#### 2.1.2. Matrix Effects

During analyte electrospray ionisation (ESI), matrix effects can cause a change in the response of the analyte, either negative (ion suppression) or positive (signal enhancement). Matrix effects were evaluated by comparing the slope of calibration curves obtained from standards prepared in solvent or spiked into *P. pinaster* stem matrix after extraction (Table 2). Results showed that the matrix has little influence on the response of most phytohormones, with slightly increasing response for BA (+12%), GA_9_ (+2%), IBA (+15%), IBA-ME (+7%), iP (+4%), Zea (+11%) and ZeaR (+16%), and small decreases in response for ABA (–13%), and IAA-ME (–0.1%). For the remaining phytohormones, stronger matrix effects were observed, with increased response for GA (+125%) and JA (+67%) and ion suppression for IAA (–39%), JA-ME (–85%) and SA (–24%), showing that components present in the matrix can influence the response of these analytes.

#### 2.1.3. Limit of Detection (LOD) and Limit of Quantification (LOQ)

The LOD (i.e., the lowest analyte concentration that can be detected in a sample) and LOQ (i.e., the lowest analyte concentration that can be quantitatively determined) were determined from a solution of a standard mixture prepared in solvent and in *P. pinaster* stem matrix (Table 2). The LOQ was established as the lowest point of the calibration curve. Cytokinins (iP, Zea and ZeaR) showed the lowest LODs (0.001–0.005 ng/mL) and LOQs (0.01–0.05 ng/mL in solvent and 0.1–0.5 ng/g in matrix). All other LODs ranged from 0.1 to 50 ng/mL and LOQs ranged from 0.5 to 100 ng/mL in solvent and 5–100 ng/g in matrix.

#### 2.1.4. Analytical Recoveries

Analytical recoveries measure the ability of the extraction protocol to extract the analyte from the matrix under study, and simultaneously, assess the influence of the extraction procedure and of the matrix. At high and medium concentrations, recoveries were high (70.8–100.5%), but for the lowest concentration (i.e., closer to the LOQ), recoveries ranged between 52.9% and 81.9%. The lowest analytical recovery was obtained for IAA, at the lowest concentration (Table 3).

#### 2.1.5. Method Precision

The precision of the LC-QqQ-MS/MS method describes the random error of the analytical method and is measured at three levels (repeatability or instrument precision, intra- and interday precision) using three concentration levels (high, medium and low). Overall, for repeatability and intraday precision, the relative standard deviation (RSD) values of peak areas were lower than 16% and 17%, respectively. Interday precision of peak area ratios showed RSD values lower than 25% (Table 3).

### 2.2. P. pinaster Early Hormonal Defence Responses to PWN Infection

#### 2.2.1. Pine Wilt Disease Progression in PWN-Inoculated *P. pinaster* Plants

**Figure 1:**
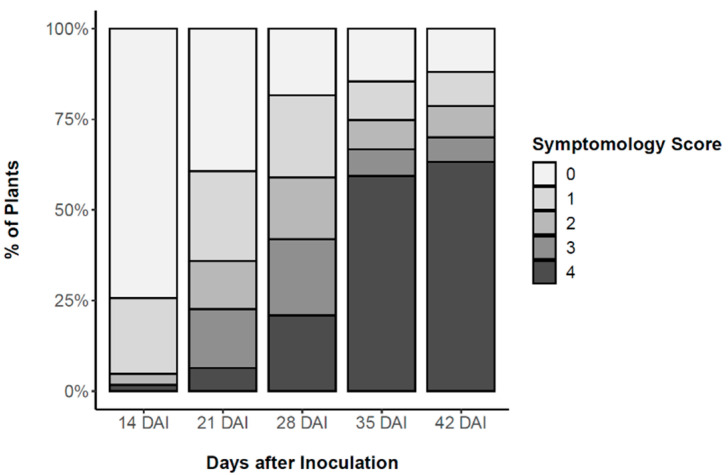
Weekly pine wilt disease (PWD) progression in *Pinus pinaster* half-sib plants after inoculation with the pinewood nematode (PWN) *Bursaphelenchus xylophilus*, according to a five-level symptomology score based on the % of needles being yellow or brown; namely, 0 (0%), 1 (1–25%), 2 (26–50%), 3 (51–75%), and 4 (76–100%).

To assure homogeneity of the 2-year-old *P. pinaster* plants, height and basal stem diameter were measured before inoculation. Plant height averaged 46.6 ± 7.5 cm and diameter averaged 4.7 ± 1.0 cm. After inoculation with the PWN, external symptoms of PWD progression were assessed weekly according to a five-level symptomology score level from 0 (no external symptoms) to 4 (all needles turned brown and wilted) (Figure 1). This symptomology score was used to distinguish between resistant and susceptible plants, harvested 24, 48 and 72 h after inoculation (HAI) to study early the role of phytohormones in defence responses. The first PWD external symptoms were observed 14 days after inoculation (DAI), when needles of symptomatic-inoculated plants started to fade to a greyish colour (needle discolouration). PWD symptoms progressed rapidly during the following days as needles wilted and turned to a yellowish and brown colour. At 35 DAI, 60% of the plants were scored a level 4, i.e., showed more than 75% of needles being desiccated and brown. A 15% survival was determined and plants showing no external symptoms were considered resistant to PWN infection. Results from the logistic regression model showed that the effects of plant height and plant diameter on plant survival were not statistically significant (failed to reject the null hypothesis, *p* > 0.05).

#### 2.2.2. Quantification of Phytohormones in *P. pinaster* Plants in Response to PWN Infection

**Figure 2:**
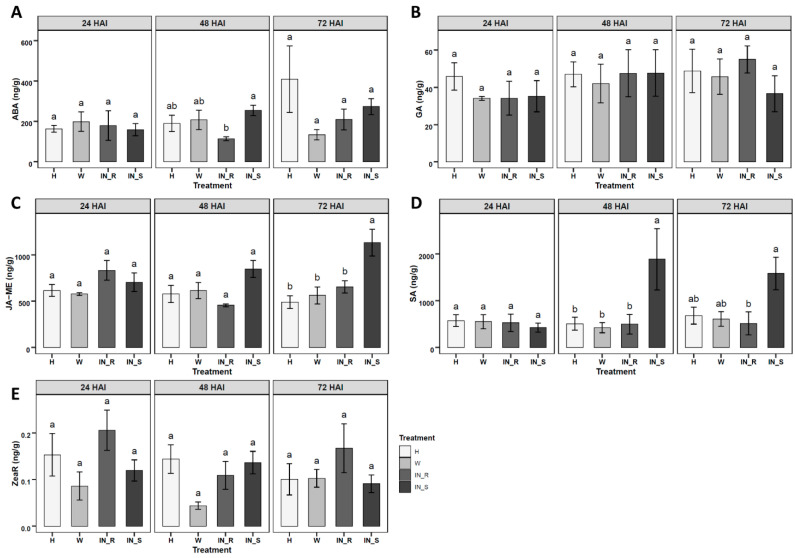
Quantification of phytohormones in stem tissues of 2-year-old *Pinus pinaster* half-sib plants (ng/g), 24, 48 and 72 h after inoculation (HAI) with the pinewood nematode (PWN) *Bursaphelenchus xylophilus*; namely, (**A**) abscisic acid (ABA), (**B**) Gibberellic acid (GA), (**C**) jasmonic acid methyl ester (JA-ME), (**D**) salicylic acid (SA), (**E**) *trans*-zeatin riboside (ZeaR). Plant treatments include healthy non-inoculated (H), mock-inoculated/wounded (W), PWN-inoculated resistant (IN_R) and PWN-inoculated susceptible (IN_S). Each bar represents the mean ± SE (error bar) of five independent biological replicates. Different letters above error bar indicate significant differences calculated using Tukey HSD test for multiple comparisons (*p* < 0.05). Absolute levels for all phytohormones can be found in Table A1.

The validated LC-QqQ-MS/MS method was applied to study changes in the absolute levels of phytohormones in stem tissues of 2-year-old *P. pinaster* plants, with contrasting responses to PWN infection. Five phytohormones were quantified in stem tissues of *P. pinaster*, namely ABA, GA, JA-ME, SA, and ZeaR, 24, 48 and 72 h after inoculation (HAI) (Figure 2, Table A1). ABA significantly decreased in resistant inoculated plants at 48 HAI, when compared to susceptible plants (Figure 2A). JA-ME showed a significant increase in susceptible plants at 72 HAI, when compared to all other treatments (non-inoculated, mock-inoculated and resistant-inoculated plants) (Figure 2C). SA showed a significant increase in susceptible plants at 48 HAI, when compared to the remaining treatments, and also a significant increase at 72 HAI, in relation to resistant plants (Figure 2D). GA and ZeaR showed no significant variation in inoculated *P. pinaster* stem tissues, among treatments for all time points (Figure 2B,E). The remaining nine phytohormones (BA, GA_9_, IAA, IAA-ME, IBA, IBA-ME, JA, iP, Zea) were detected in levels below the LOQ, and therefore were not quantified.

## 3. Discussion

### 3.1. LC-QqQ-MS/MS Analytical Method Validation

Due to their low abundance and highly diverse chemical structures, phytohormones are challenging metabolites to quantify and extraction protocols can involve several purification steps. To overcome this challenge, this work describes the development and validation an LC-QqQ-MS/MS method to quantify 14 target phytohormones in the complex matrix of *P. pinaster* stem tissues. Tissues of tree species are complex matrices known to contain interferents such as resin acids, oleoresins, terpenoids, celluloses, and lipids that can contribute to matrix effects during the quantification of phytohormones [38]. In addition, reports of analytical methods for quantification of phytohormones that are validated for forest tree tissues are scarce and technical difficulties have been reported in the literature when applying analytical methods originally developed and validated for other plant species. Pan et al. [41] described for the first time a relatively simple liquid–liquid extraction (LLE) protocol, using 2-propanol: H_2_O:HCl (2:1:0.002, *v/v/v*) and dichloromethane for the simultaneous quantification of multiple classes of phytohormones in Arabidopsis leaf extracts. However, when applied to the extraction of phytohormones from forest tree tissues some constraints have been reported; namely, no phase separation could be observed after centrifuging, leading to the need for additional clean-up steps (e.g., solid phase extraction, SPE) [38]. To avoid this time-consuming step, in this work, phytohormones were extracted using methanol: H_2_O (7:3, *v*/*v*) without further purification steps, adapted from an extraction protocol previously described by Cao et al. [32] for barley roots matrix.

Methanol has gradually become the preferred organic solvent for extraction of phytohormones due to its low molecular weight and small size, thus being able to penetrate easily into plant cells during extraction [29]. A previous study showed that, when compared to non-polar organic solvents, methanol and water mixtures provided higher phytohormone extraction efficiency using dry and fresh plant material. Furthermore, the comparison of several different ratios of methanol: H_2_O showed that a 7:3 (*v*/*v*) ratio proved the most efficient for extraction of multiple phytohormones, leading to a lower chlorophyll content in the final extract and without needing further purification steps [35].

Among the methods available for quantification of phytohormones, MS technology is the most powerful due to its high sensitivity and selectivity. Gas chromatography (GC) and LC have been used for quantification of phytohormones since the early 1970s [29]. The analysis of phytohormones using GC-MS requires sample derivatisation prior to analysis in order to enhance volatility and improve the thermal stability of the compounds [29,30,34]. However, derivatisation involves several steps during sample preparation, which can compromise yields and also makes the analysis of phytohormones by GC-MS time consuming. Additionally, not all compounds can be volatilised, and high temperatures from the GC injector and columns can degrade thermally labile compounds. To address these issues, LC-MS/MS has emerged as the method of choice for quantification of phytohormones, in particular, use of the LC-QqQ-MS system [34,41,42,43,44]. Tracking a specific precursor-to-product ion transition for each phytohormone, the SRM technique minimises noise, and maximises sensitivity and selectivity.

In this study, acetic acid was used as the weak acid modifier. Acetic acid was reported to enhance the negative-ion ESI response of phenolic compounds at low concentrations [45], and also produces overall favourable responses for positive ion-producing compounds [46]. Acetic acid has been widely used as a mobile phase additive in LC-MS/MS methods for the quantification of multiple phytohormones [37,43,47,48].

The total run time of 6.6 min is a major advantage of this analytical method. Most available analytical methods for the quantification of phytohormones in plant matrices, including forest tree tissues, rely on longer run times (around 17–25 min) [35,36,37,38,44,49]. Forest tree research is usually performed on a large scale, mostly due to the genetic variability between and within species, and for that reason can benefit greatly from a shorter LC-MS analysis time.

In the plant sciences, due to the lack of a blank matrix (i.e., a matrix containing none of the analytes under study) or certified reference materials (CRMs), several adjustments have to be performed in some key steps of the analytical method validation, such as measuring analytical recoveries of spiked metabolites as a measure of accuracy [50]. In this case, the recovery of the method is determined by comparing the response of analytes spiked into a biological sample prior to the sample extraction with the response of the same metabolites spiked after extraction. When preparing calibration standards in plant matrix, in this study, the correction of the endogenous amounts of phytohormones detected in pooled plant matrix were also taken into account for the method validation using the background subtraction approach [49]. Background subtraction is widely used in bioanalytical method validation in the absence of a blank matrix because it (i) allows to use the same biological matrix as in the samples under study, (ii) does not depend on the availability of the significant amounts of matrix needed for performing standard additions, and (iii) does not require large amounts of expensive labelled standards as surrogate analytes. Other methods include using a surrogate matrix, or surrogate analyte or a standard addition method [39]. Although not directly affecting the slope of the calibration curve, the presence of the endogenous analyte in the biological matrix may be responsible for a larger intercept value [36] and is often ignored in most method validation studies for the analysis of phytohormones.

Strong matrix effects have been reported for several phytohormones in tree matrices; namely, ion suppression for GA_7_, SA and ABA (–43, –22 and –19%, respectively) and ion enhancement for GA4, JA, BA, Zea (+25%) and ZeaR (an enhancement of 4-fold) [38]. In the study by Trapp et al. [35] the evaluation of matrix effects revealed that *Arabidopsis thaliana* (L.) Heynh. and *Citrus sinensis* (L.) Osbeck matrices (leaves) influenced phytohormone responses very differently; namely, for IAA (–31 and –16%), JA (+7 and +147%), JA-Isoleucine (–25 and +86%), SA (+46 and –4%), and the jasmonate precursor 12-oxo-phytodienoic acid (OPDA, –87 and –32%), respectively. The comparison of different matrix effect studies showed that the effects of the biological matrix on quantitative methods strongly depends on both analyte and matrix composition. In tissues of tree species (e.g., stems) the presence of resin acids, oleoresins, terpenoids, celluloses, and lipids can contribute to strong matrix effects [38]. Thus, and particularly when the matrix has influence in the response of the target analyte(s), the most reliable way to evaluate matrix effects on quantitative results is by using calibration curves fully developed in the presence of the matrix [35].

LOD and LOQ values were of comparable order of magnitude to results from other phytohormone method validation studies [32,35]. Similar RSD values have been reported [35,37,38,44], and also tend to increase for low-level concentrations (closer to LOQ). The lowest analytical recovery was obtained for IAA, at the lowest concentration. This might be explained due to the labile nature of IAA, as it is reported to be unstable under certain conditions; namely, heat, light, the presence of salts and even dark conditions [51]. This emphasises the importance of performing analytical recovery studies over the full range of concentrations of the calibration standards as well as performing the whole extraction protocol under optimal conditions (e.g., dark and 4 °C). Results for the analytical recoveries demonstrate that the targeted low-abundance phytohormones can be successfully recovered from the matrix, and be accurately quantified in *P. pinaster* stem tissues following LC-QqQ-MS/MS analysis.

### 3.2. P. pinaster Early Hormonal Defence Responses to PWN Infection

PWN infection led to a significant increase in SA (48 and 72 HAI) and JA-ME (72 HAI) concentration in susceptible *P. pinaster* plants, in relation to the resistant phenotype. To date, only transcriptomic approaches have estimated the hormonal defence response of resistant and susceptible phenotypes of *Pinus* spp. after PWN infection. Transcriptomic profiling in PWN infected *Pinus massoniana* Lamb. revealed a smaller number of differentially expressed genes in the resistant phenotype, when compared to the susceptible plants [18]. In addition, transcriptional differences between resistant and susceptible *Pinus thunbergii* Parl., revealed that gene expression of pathogenesis-related (PR) proteins were induced more rapidly (24 HAI), and with a higher magnitude, in susceptible trees, whereas this induction occurred more slowly and weakly in resistant trees [52]. SA accumulation has a key role in HR [53], which in PWN infection includes rapid cell death (parenquima), production of toxins, and leakage of oleoresin into the tracheids [54]. The observed SA accumulation in this study is in agreement with previous studies on *P. thunbergii,* suggesting that after PWN invasion, a series of HRs are rapidly triggered in susceptible trees, and ultimately lead to tree death. In contrast, a moderate HR together with the upregulation of PR genes and cell wall-related genes to restrict PWN migration could be a more effective defence against PWN infection [52].

In the regulation of the plant defence responses, most PR proteins are induced through the action of the signalling metabolites SA, JA or ET [55]. SA is generally involved in the activation of defence responses against biotrophic and hemi-biotrophic pathogens, whereas JA and ET are responsible for defence responses against necrotrophic pathogens and herbivorous insects. However, the PWN feeds from multiple sites and is considered a migratory endoparasitic nematode. These nematodes use the live tree tissues for feeding and multiplying (propagative cycle), and once the host is killed, nematodes feed on the fungi colonising dead or dying trees (dispersal cycle) [56]. Moreover, previous studies have also shown that during the propagative cycle, PWN infection can induce the production of reactive oxygen species (e.g., peroxide oxide) in pine hosts, which leads to the accumulation of SA [57].

The JA and ET signalling pathways operate synergistically in the regulation of defence related genes, whereas an antagonist crosstalk is often observed between SA and JA/ET signalling pathways [27]. Even though JA could not be quantified (i.e., below LOQ), the significant increase in its conjugated form JA-ME (72 HAI) and in SA (48 and 72 HAI) in susceptible plants indicates that the accumulation of these defence phytohormones did not lead to a higher tolerance of *P. pinaster* to PWN infection. Moreover, the significant increase only in SA at 48 HAI in susceptible plants, when compared to resistant and control plants, can suggest an antagonist crosstalk between SA and JA-ME. Similarly, a negative crosstalk between SA and JA-ME has been reported in other plant defence responses [58].

In this study, a lower ABA concentration was observed in resistant *P. pinaster* plants at 48 HAI than in susceptible plants. ABA is known to stimulate short-term responses like closure of stomata, resulting in maintenance of water balance or protection against invading leaf pathogens [59]. In addition, the positive or negative regulatory role of ABA on plant defence depends on the plant–pathogen interaction [60]. Furthermore, ABA is suggested to undergo crosstalk with other signalling pathways; namely, SA-, ET- and sugar-mediated signalling pathways. The downregulation of the ABA pathway has been linked to increased infection tolerance, although changes are much smaller when compared to those of SA or JA [61]. Similarly, the observed ABA decrease in resistant plants shortly after PWN infection could be a coping mechanism to regulate plant defence responses, thereby contributing to *P. pinaster* tolerance to PWN infection.

## 4. Materials and Methods

### 4.1. Chemicals and Reagents

Water and solvents used for extraction and chromatography and mobile phase additive acetic acid were all HPLC grade and purchased from Fisher Scientific (Loughborough, UK). Phytohormone standards (PH) and stable isotope-labelled internal standards (IS) were purchased from a range of suppliers (Supplementary Table S1). Authentic standard compounds included ABA, BA, GA, GA_9_, IAA, IAA-ME, IBA, IBA-ME, JA, JA-ME, iP, SA, Zea, and ZeaR. IS included: d_6_-ABA, d_5_-BA, d_2_-GA_9_, d_5_-IAA, d_5_-IAA-ME, d_4_-SA, and d_5_-Zea. Non-labelled internal standards included (±)-9,10-dihydrojasmonic acid (DHJA), and (±)-9,10-dihydrojasmonic acid methyl ester (DHJA-ME).

### 4.2. Plant Material

*Pinus pinaster* seeds were obtained from a top genetically ranked plus tree for PWD resistance (family 152) belonging to a breeding population, and ranked on the 1st EBLUP position out of 96. The mass selection program was performed at “Herdade da Comporta” (38°21′28.52″N; 8°45′49.89″W) in southern Portugal [9,62]. For cold-wet stratification, seeds were placed on a double layer of filter paper soaked in sterile distilled water, in Petri dishes sealed with Parafilm^®^, and kept in the dark for 3 weeks at 4 °C. Stratified seeds were germinated in forestry trays (Cetap 54-universal), in greenhouse conditions, at the Instituto Nacional de Investigação Agrária e Veterinária (INIAV, Oeiras, Portugal). *P. pinaster* half-sib plants were grown under natural daylight, and the greenhouse was equipped with a cooling system (for a maximum and minimum temperature of 28 and 15 °C, respectively, and average humidity 65%), and an automatic sprinkler irrigation system set for 5 min for 48 h during the winter, and 3 min for 24 h during the summer. When *P. pinaster* plants reached 1-year-old, 24 plants were harvested to perform the optimisation of the extraction protocol and LC-QqQ-MS/MS analytical method validation. The remaining plants continued to grow for a further year for the PWN inoculation experiment and quantification of phytohormones in response to PWN infection.

### 4.3. LC-QqQ-MS/MS Analytical Method Validation

#### 4.3.1. Standard Stock Solutions and Quality Controls

PH and IS stock solutions were individually prepared in methanol at a concentration of 1.0 mg/mL and 50 μg/mL, respectively, and stored for no longer than 3 months at –20 °C prior to use. Quality control samples (QCs) consisting of a mixture of all PHs were prepared in methanol: H_2_O (70:30, *v*/*v*) at three concentration levels (low, medium, and high) along the whole linearity range defined by the calibration curves. The high-level QC solution contained 5000 ng/mL of BA, JA; 1500 ng/mL of ABA, IAA, IAA-ME, IBA, IBA-ME, GA, GA_9_, JA-ME; and 50 ng/mL of iP, SA, Zea, ZeaR. Medium level QC solutions contained 2000 ng/mL of BA, JA; 500 ng/mL of ABA, IAA, IAA-ME, IBA, IBA-ME, GA, GA9, JA-ME; and 35 ng/mL of iP, SA, Zea, ZeaR. Low level QC solutions contained 500 ng/mL BA, JA; 100 ng/mL of GA, GA_9_, JA-ME; 50 ng/mL of ABA, IAA, IAA-ME, IBA, IBA-ME; 5 ng/mL of iP, SA, Zea and ZeaR. These QC solutions were used in method precision and analytical recovery determinations.

#### 4.3.2. Extraction of Phytohormones

The extraction of phytohormones was optimised using a protocol adapted from Cao et al. [32]. Briefly, 50 mg (FW) of finely homogenised frozen stem tissues from 1-year-old *P. pinaster* half-sib plants (pool of 24 independent plants) were weighed into 2.0 mL safe-lock microfuge tubes, and kept in liquid nitrogen to avoid thawing. To each tube, 1.5 mL of ice-cold methanol: H_2_O (70:30, *v*/*v*), including a fixed amount of IS (2000 ng/mL of d_5_-BA and DHJA, 500 ng/mL of d_6_-ABA, d_5_-IAA, d_5_-IAA-ME, d_2_-GA_9_ and DHJA-ME, and 35 ng/mL of d_5_-Zea and d_4_-SA) were added and briefly vortex-mixed. The mixture was incubated on a shaker for 30 min (in the dark), at 500 rpm and 4 °C, followed by centrifuging at 14,000 g for 5 min, at 4 °C. The supernatant (~1.4 mL) was transferred to a new tube and evaporated to dryness in a vacuum concentrator (GeneVac EZ-2, UK) using the low boiling point method, for 3 h. Samples were reconstituted in 50 μL of methanol: H_2_O (70:30, *v*/*v*), briefly sonicated, vortexed, and centrifuged at 14,000× *g*, for 5 min and at 4 °C, and subsequently analysed and quantified by LC-QqQ-MS/MS.

#### 4.3.3. LC-QqQ-MS/MS Instrument Setup

LC analyses were carried out on a Dionex Ultimate 3000™ HPLC system (Dionex Softron, Germany). LC separations were performed on an XSelect CSH C18 HPLC column (130 Å, 3.5 µm, 2.1 × 100 mm; Waters Corporation, Milford, MA, USA) maintained at 40 °C. The column flow rate was set at 0.5 mL/min, and the injection volume was 4 μL. The LC mobile phase consisted of (A) water containing 0.1% acetic acid and (B) acetonitrile containing 0.1% acetic acid. The optimal LC elution profile was 0–1.22 min 10% B; 1.22–4.64 min, 10–100% B; 4.64–5.6 min 100% B; 5.6–5.62 min 100–10% B; 5.62–6.6 min 10% B, allowing the column to equilibrate for 0.98 min. MS/MS experiments were performed on a TSQ Endura™ QqQ-MS/MS (Thermo Scientific, San Jose, CA, USA) equipped with an electrospray ionisation (ESI) source, operating in either negative or positive ionisation mode, according to the ionisation efficiency determined for each analyte. Measurements were carried out using the following ionisation parameters: source voltage for positive and negative analyses: 3500 and 2500 V, respectively; sheath gas: 50 (arbitrary units); auxiliary gas: 15 (arbitrary units); sweep gas 2 (arbitrary units); ion transfer tube temperature: 350 °C; vaporiser temperature: 400 °C, cycle time: 0.5 s; CID gas: 1.5 mTorr. SRM parameters for each target analyte were optimised using the Thermo Scientific Automated Compound Optimisation (Thermo Xcalibur software version 4.0.27.10, Thermo Scientific, San Jose, CA, USA) by directly infusing into the mass spectrometer individual standard solutions of each compound (40 μg/mL) prepared in methanol at a flow rate of 0.1 mL/min. Full-scan data acquisition was performed using the first mass analyser (Q1) by scanning from *m*/*z* 50 to 1000. All data were collected and processed using Thermo Xcalibur software version 4.0.27.10 QualBrowser and QuanBrowser (using Genesis peak detection algorithm) software (Thermo Scientific, San Jose, CA, USA).

#### 4.3.4. Calibration Curves and Linearity

Calibration standards were prepared both in solvent (methanol: H_2_O, 70:30, *v*/*v*) and in extract of the pooled 1-year-old *P. pinaster* matrix, using three different mixed standard solutions: standard solution A containing BA and JA at 50, 100, 500, 2000, 3500 and 5000 ng/mL; standard solution B containing ABA at 5, 50, 100, 500, 1000, 1500 ng/mL, GA at 100, 250, 500, 1000, 1500 ng/mL and IAA, IAA-ME, IBA, IBA-ME, GA_9_, JA-ME at 50, 100, 500, 1000, and 1500 ng/mL; standard solution C containing iP, SA, Zea, ZeaR at 0.1, 1, 5, 20, 35, 50 ng/mL (the last concentration only for iP, Zea and ZeaR). All standard solutions contained a fixed amount of IS, namely 2000 ng/mL of d_5_-BA and DHJA for standard solution A, 500 ng/mL of d_6_-ABA, d_5_-IAA, d_5_-IAA-ME, d_2_-GA9 and DHJA-ME for standard solution B and 35 ng/mL of d_5_-Zea and d_4_-SA for standard solution C. To establish the calibration range for each phytohormone, endogenous phytohormone levels were estimated by comparing peak areas of endogenous levels with peak areas of known amounts of the corresponding IS spiked into the pooled *P. pinaster* matrix. Five-point standard curves were then established, with each point corresponding to a mean value of at least three independent measurements. Due to the lack of a blank matrix, for calibration standards prepared in pooled plant matrix, correction for endogenous levels of detected phytohormones was performed using a background subtraction approach, i.e., the endogenous phytohormone peak areas were subtracted from the total peak area obtained after the standard was spiked into the pooled plant matrix [54]. Both determination coefficients (*R*^2^) and residual plots were used to evaluate the linearity and homoscedasticity of the calibration for each phytohormone. The quantification of phytohormones in *P. pinaster* stem tissues was determined from the measured peak area ratios of endogenous phytohormones to the corresponding IS, using the established standard calibration curves prepared in matrix.

#### 4.3.5. Matrix Effects

Matrix effects (*ME*) were quantitatively evaluated by comparing the signal response of a target analyte in the absence of matrix (standard solution) with the signal response of the analyte spiked at the same concentration into the 1-year-old *P. pinaster* matrix extract [63]. The percent *ME* (%) was calculated as in Equation (1):

(1) $$ME\;\left(\%\right)=\;\left(\frac{slope\;of\;calibration\;curve\;in\;matrix}{slope\;of\;calibration\;curve\;in\;solvent}-1\right)\;\times 100$$

where *ME* = 0% indicates no matrix effect, negative values indicate ion suppression, and positive values indicate signal enhancement. For this purpose, the slopes of the calibration curves were used.

#### 4.3.6. LOD and LOQ

LOD and LOQ were determined using Thermo Xcalibur version 4.0.27.10 QuanBrowser (Thermo Scientific, San Jose, CA, USA) and the built-in Genesis peak detection algorithm. The LOD and LOQ were estimated from the lowest concentration from serial dilutions of authentic standards in solvent, and in the 1-year-old *P. pinaster* matrix extract, that gave a signal-to-noise (S/N) ratio, of 3 and 10, respectively. S/N calculations were based on the ratio of the peak height to the noise in the baseline.

#### 4.3.7. Analytical Recoveries

Due to the lack of a blank matrix or CRMs, analytical recoveries are evaluated as a measure of the accuracy of the method [50]. Analytical recoveries were determined using QC samples at three concentration levels (low, medium and high), by comparing the amount recovered of each phytohormone spiked into the extraction solvent (methanol: H_2_O, 70:30, *v*/*v*) containing pooled 1-year-old *P. pinaster* matrix at the beginning of the extraction, with the amount recovered of each phytohormone spiked after extraction [35,39,50] as in Equation (2):

(2) $$Recovery\;\left(\%\right)=\;\left(\frac{analyte\;peak\;area\;spiked\;at\;the\;beginning\;of\;extraction}{analyte\;peak\;area\;spiked\;after\;extraction}\right)\;\times 100$$

Thus, the analytical recovery is expressed in terms of a percentage of each recovered phytohormone spiked into the extract of the *P. pinaster* matrix.

#### 4.3.8. Method Precision

The precision of the method was measured at three levels; namely, repeatability (or instrument precision), intra- and interday precision [39,50,64], using QC samples at three different concentration levels (high, medium and low). Instrument precision was assessed from six consecutive injections of the same QC sample, and expressed as the relative standard deviation (RSD, %) of peak areas for each compound. Intraday precision was determined by injecting the same QC sample three times within a single day, and expressed as the RSD of analyte to IS peak area ratio. Interday precision was determined over three different days (1st, 2nd and 5th day), and expressed as the RSD of analyte to IS peak area ratio.

### 4.4. Experimental Design, PWN Inoculation and Sampling Procedure

*Bursaphelenchus xylophilus* isolate, Bx013.003 (GenBank database (NCBI) accession number MF611984.1) was obtained from the INIAV’s Nematology Laboratory, Oeiras, Portugal.

The experimental design layout consisted of a completely randomised design, with two factors: inoculation and time after inoculation. Inoculation had four levels (i.e., noninoculated, mock-inoculated (i.e., wounded), susceptible, and resistant PWN-inoculated 2-year-old *P. pinaster* half-sib plants), and time after inoculation had three levels (24, 48 and 72 HAI). For a total of 324 plants, height and basal stem diameter of each plant were measured before inoculation, using a marked scale and a digital caliper (Mitutoyo CD-15DCX, Mitutoyo Corp., Kawasaki, Japan).

For the inoculation process, needles were manually removed from an area of ca. 5 cm in the upper part of the stem of each plant, and superficial longitudinal incisions were performed with a sterile razor blade. A sterilised piece of cotton was placed and fixed with Parafilm^®^, and a suspension with an estimated number of 500 PWNs (at various stages of development) was applied with a micropipette. The cotton was gently covered with Parafilm^®^ to prevent the inoculum from drying. A mock inoculation was performed by replacing the PWN suspension with sterile water. A portion of the stem (ca. 5 cm) above the inoculation area was collected 24, 48 and 72 HAI, as well as the corresponding stem portion in non-inoculated plants. Samples were immediately placed in liquid nitrogen (shock freezing) and stored at –80 °C until processing. The remaining part of the *P. pinaster* plants were maintained for 42 DAI for PWD symptoms observation and classification (i.e., susceptible or resistant plants). External symptoms were assessed weekly 14, 21, 28, 35 and 42 DAI on inoculated plants, in a symptomology score established according to percentage of discoloured and wilted needles in susceptible inoculated plants; namely, 1 (1–25%), 2 (26–50%), 3 (51–75%), and 4 (76–100%). According to the obtained plant classification, five biological replicates for each treatment were randomly selected per time point.

In this study, resistance to PWN infection was considered when inoculated plants showed no PWD external symptoms (0%), whereas the symptomatic plants were considered susceptible to PWN infection. As the definition of resistant and tolerant plants cannot be differentiated based solely on external symptoms, plants considered as resistant in this study include their ability to defend or withstand the pathogenic attack, as in Carrasquinho et al. [9].

During the course of the experiment, the average day/night air temperature and relative humidity was 25/20 °C and 61/71%, respectively.

### 4.5. Extraction and Quantification of Phytohormones in PWN-Inoculated P. pinaster

Extraction of phytohormones was performed as described in Section 4.3.2. Each tube contained 50 mg (FW) of finely homogenised 2-year-old *P. pinaster* stem powder in 1.5 mL of ice-cold methanol: H_2_O (70:30, *v*/*v*), including a fixed amount of IS (5000 ng/mL of d_5_-IAA and DHJA-ME and 200 ng/mL of d_6_-ABA, d_5_-BA, d_2_-GA_9_, d_5_-IAA-ME, DHJA, d_5_-Zea, and d_4_-SA). Phytohormones were analysed and quantified using the LC-QqQ-MS/MS method described in Section 4.3.3. Calibration ranges for calibration curves were chosen based on the estimated concentration of phytohormones in 2-year-old *P. pinaster* stem samples from the inoculation experiment; namely, 20, 100, 500, 2500, 5000, 10,000 ng/mL for GA; 10, 50, 250, 1250, 2500, and 5000 ng/mL for SA; 1, 5, 25, 125, 250, 500 ng/mL for ABA; 0.1, 0.5, 2.5, 12.5, 25, 50 ng/mL for ZeaR (Supplementary Table S2).

### 4.6. Statistical Analyses

Statistical analyses were performed in R and R Studio (Boston, MA, USA) software [65,66]. R Packages used to perform statistical analysis include “agricolae” [67], and “gplots” [68].

To analyse plant survival, a logistic regression, i.e., a generalised linear model (glm) with logit link function and binomial error distribution, was fitted. The model considered the effects of plant height and plant diameter as predictors in the survival outcome. Binned residual plot was used to confirm the fit of the regression model.

For phytohormone analyses, one-way ANOVA at a 95% confidence level was used to assess differences between treatments per time point, and multiple comparison analysis was performed using Tukey’s HSD test. The wound effect was assessed by comparing mock-inoculated with non-inoculated controls. PWN infection responses were evaluated by comparing PWN inoculated with mock-inoculated plants.

## 5. Conclusions

This work reports the development and validation of an LC-QqQ-MS/MS method for the targeted analysis of 14 phytohormones from six major classes; namely, ABA, AXs, CKs, GAs, JAs and SA, using the highly selective SRM mode. The current methodology offers advantages over existing methods for forest tree tissues, namely shorter analysis time (6.6 min) and simpler sample preparation. The cold extraction protocol using methanol: H_2_O (70:30, *v*/*v*) is simple, fast, without the need for a pre-LC purification step, and can be used to avoid the technical difficulties previously found when performing LLE of phytohormones from tree tissues. This analytical method was further applied to quantify these metabolites in 2-year-old *P. pinaster* stem tissues, after inoculation with the PWN. The results showed an increase in SA and JA-ME in susceptible-inoculated plants but not in resistant plants, thus supporting the hypothesis of an inefficient activation of a hypersensitive response (HR) that leads to tree death, whereas resistant plants showed a more moderate HR. The simple and fast methodology for the quantification of phytohormones presented in this work greatly facilitates large-scale studies and can be further used as a tool for phytohormone-assisted selection of progeny in nurseries and phenotyping or breeding programs.

## Figures and Tables

**Table 1:** Selected reaction monitoring (SRM) transitions of 14 target phytohormones (PH) and corresponding internal standards (IS) determined using a Thermo Scientific™ TSQ Endura system™. PH: phytohormone; IS: internal standard; IM: electrospray ionisation mode (ESI− or ESI+); *t*_R_: retention time (min); SRM: selected reaction monitoring (* indicates the quantification transition); CE: ‘collision energy’ setting (V). For PH and IS abbreviations see Supplementary Table S1.

Analyte (PH)	IM	*t*_R_ (min)	SRM	CE (V)	Analyte (IS)	IM	*t*_R_ (min)	SRM	CE (V)
ABA	–	4.08	263.1 > 153.1 *	10.354	d_6_-ABA	–	4.07	269.1 > 159.1	10.354
			263.1 > 219.1	13.438					
BA	–	3.80	121.2 > 77.2 *	11.719	d_5_-BA	–	3.78	126.2 > 82.2 *	10.253
			121.2 > 93.0	10.809				126.2 > 125.6	35.129
GA	–	3.46	345.2 > 239.1 *	15.562	d_2_-GA_9_	–	5.03	317.2 > 273.2	21.174
			345.2 > 221.1	25.421					
GA_9_	–	5.02	315.2 > 271.2 *	21.022	d_2_-GA_9_	–	5.03	317.2 > 273.2	21.174
			315.2 > 253.1	27.242					
IAA	–	3.84	174.1 > 130.1 *	10.253	d_5_-IAA	–	3.83	179.1 > 135.2 *	10.253
			174.1 > 128.1	19.152				179.1 > 133.1	19.404
IAA-ME	+	4.45	190.2 > 130.1 *	10.253	d_5_-IAA-ME	+	4.45	195.1 > 134.1 *	10.253
			190.2 > 103.2	35.989				195.1 > 135.1	13.640
IBA	+	4.35	204.2 > 130.1 *	25.876	d_5_-IAA-ME	+	4.45	195.1 > 134.1 *	10.253
			204.2 > 144.1	22.893				195.1 > 135.1	13.640
IBA-ME	+	4.85	218.2 > 186.0 *	10.253	d_5_-IAA-ME	+	4.45	195.1 > 134.1 *	10.253
			218.2 > 130.1	27.646				195.1 > 135.1	13.640
iP	+	3.00	204.2 > 136.0 *	15.511	d_5_-Zea	+	0.60	225.3 > 137.1 *	17.888
			204.2 > 148.0	10.253				225.3 > 148.0	15.360
JA	–	4.39	209.1 > 59.3 *	14.096	DHJA	–	4.59	211.2 > 59.3 *	14.096
			209.1 > 165.1	10.253				211.2 > 167.1	17.685
JA-ME	+	4.92	225.2 > 151.1 *	12.073	DHJA-ME	+	4.80	227.2 > 135.1 *	10.253
			225.2 > 133.1	14.803				227.2 > 153.1	12.225
SA	–	4.07	137.2 > 93.2 *	16.219	d_4_-SA	–	4.10	141.1 > 97.2 *	17.180
			137.2 > 65.3	28.455				141.1 > 69.3	29.719
Zea	+	0.61	220.2 > 136.1 *	17.180	d_5_-Zea	+	0.60	225.3 > 137.1 *	17.888
			220.2 > 148.0	14.753				225.3 > 148.0	15.360
ZeaR	+	0.63	352.2 > 220.0 *	19.000	d_5_-Zea	+	0.60	225.3 > 137.1 *	17.888
			352.2 > 136.1	31.893				225.3 > 148.0	15.360

**Table 2:** Method validation parameters of 14 target phytohormones (PH): concentration range (ng/mL) used for linearity determined both in solvent (methanol: H_2_O, 70:30, *v*/*v*) and in 1-year-old *Pinus pinaster* stem matrix, matrix effects (%), limit of detection (LOD), limit of quantification in solvent (LOQ_S_) and in matrix (LOQ_M_). For analyte abbreviations see Supplementary Table S1.

Analyte (PH)	Conc. Range (ng/mL)	Linearity (Solvent)	*R* ^2^	Linearity (Matrix)	*R* ^2^	ME (%)	LOD (ng/mL)	LOQ_S_ (ng/mL)	LOQ_M_ (ng/g)
ABA	5–1500	0.00251*x* + 0.0143	0.993	0.00218*x* + 0.0234	0.991	–13	1	5	5
BA	50–5000	0.000428*x* + 0.0969	0.996	0.000478*x* + 0.0980	0.998	+12	10	50	50
GA	100–1500	0.000053*x* − 0.0019	0.993	0.000119*x* − 0.00441	0.991	+123	5	10	50
GA_9_	100–1500	0.00182*x* + 0.0172	0.996	0.00185*x* + 0.0226	0.991	+2	5	50	100
IAA	50–1500	0.00255*x* + 0.00695	0.992	0.00155*x* + 0.00850	0.992	–41	5	50	50
IAA-ME	50–1500	0.00322*x* − 0.0398	0.990	0.00322*x* − 0.0343	0.990	–0.1	5	10	50
IBA	50–1500	0.00129*x* − 0.0231	0.994	0.00149*x* − 0.2189	0.992	+15	5	10	50
IBA-ME	50–1500	0.00461*x* − 0.117	0.996	0.00495*x* − 0.0332	0.991	+7	1	10	50
iP	1–50	0.0665*x* − 0.0222	0.996	0.0693*x* − 0.0269	0.993	+4	0.005	0.05	0.5
JA	50–5000	0.000212*x* + 0.0190	0.991	0.000355*x* + 0.0195	0.990	+67	5	50	50
JA-ME	100–1500	0.00365*x* − 0.0792	0.996	0.000522*x* − 0.0317	0.991	–85	50	50	100
SA	5–50	0.0119*x* + 0.0299	0.995	0.00901*x* + 0.176	0.991	–24	0.1	0.5	5
Zea	5–50	0.0160*x* + 0.000412	0.998	0.0177*x* + 0.000550	0.990	+11	0.001	0.01	0.1
ZeaR	5–50	0.0102*x* + 0.00246	0.999	0.0117*x* + 0.00345	0.990	+16	0.001	0.01	0.1

**Table 3:** Method validation parameters of 14 target phytohormones (PH) for three concentration levels (high, medium and low): estimated analytical recovery (%), instrument precision (relative standard deviation, RSD, %), intraday precision (RSD, %), and interday precision (RSD, %). Analytical recovery values presented as average ± standard error, *n* = 3 extraction rounds). RSD was determined for each phytohormone concentration level by multiplying the standard deviation by 100 and dividing this product by the average.

Analyte (PH)	Concentration (ng/mL)	Analytical Recovery (%) ± SE	Instrument Precision (RSD, %)	Intraday Precision (RSD, %)	Interday Precision (RSD, %)
ABA	1500	84.9 ± 4.4	2.8	2.1	11.9
	500	80.5 ± 5.9	5.3	5.8	10.7
	50	73.6 ± 5.4	14.2	8.9	22.2
BA	5000	94.1 ± 2.0	0.6	3.0	6.0
	2000	84.7 ± 5.2	0.7	0.7	4.4
	500	79.9 ± 14.8	2.7	1.8	6.6
GA	1500	83.8 ± 8.1	4.6	0.5	7.4
	500	88.5 ± 9.1	9.0	4.5	6.3
	100	63.8 ± 20.0	7.7	12.8	22.0
GA_9_	1500	97.5 ± 0.3	2.7	0.3	1.7
	500	88.3 ± 3.8	2.3	1.6	1.7
	100	81.7 ± 5.5	5.7	2.1	6.8
IAA	1500	71.2 ± 7.7	10.6	2.4	10.1
	500	70.8 ± 8.9	13.4	5.5	12.3
	50	52.9 ± 14.2	12.6	16.9	19.3
IAA-ME	1500	94.7 ± 4.0	1.4	1.7	11.7
	500	89.2 ± 2.8	2.4	1.2	10.0
	50	81.9 ± 1.9	9.9	5.8	22.9
IBA	1500	93.1 ± 8.8	4.0	4.0	16.4
	500	74.5 ± 5.6	2.4	3.9	15.9
	50	67.2 ± 13.4	8.5	3.6	22.9
IBA-ME	1500	90.6 ± 9.2	3.1	1.4	17.9
	500	89.0 ± 0.4	2.8	9.0	21.6
	50	76.8 ± 3.9	6.3	12.3	18.4
iP	50	89.5 ± 2.9	1.3	1.7	20.2
	35	83.9 ± 9.2	3.8	1.0	20.3
	5	67.9 ± 10.0	2.7	3.7	25.0
JA	5000	100.6 ± 7.5	2.4	0.3	5.6
	2000	71.1 ± 1.3	5.3	5.0	6.5
	500	67.6 ± 10.5	13.5	8.2	9.1
JA-ME	1500	90.3 ± 3.3	3.7	3.6	22.8
	500	80.0 ± 3.6	3.9	3.7	22.1
	100	71.3 ± 28.5	15.9	13.4	18.1
SA	50	92.2 ± 2.6	14.2	1.2	2.8
	35	88.9 ± 3.5	14.4	4.0	4.3
	5	70.8 ± 3.2	1.3	2.2	5.5
Zea	50	97.5 ± 3.6	2.3	1.6	3.4
	35	84.4 ± 1.2	0.9	2.1	3.9
	5	69.2 ± 17.6	2.0	3.3	4.6
ZeaR	50	92.9 ± 13.1	2.5	1.3	8.1
	35	85.6 ± 1.0	2.0	3.2	10.3
	5	66.9 ± 11.2	5.5	1.5	21.1

## Data Availability

The data presented in this study are available in supplementary material.

## Supplementary Materials

The following are available online at https://www.mdpi.com/article/10.3390/metabo11040227/s1, Table S1: Phytohormones (PH) and stable isotope-labelled internal standards (IS) used in this work, Figure S1: Selected reaction monitoring (SRM) chromatograms obtained using ESI+ and ESI− for the LC-QqQ-MS/MS separation of 14 target phytohormones (1 µg/mL), Figures S2 and S3: Residual plots for evaluating the homoscedasticity and goodness of fit of the calibration curves prepared in solvent and in 1-year-old *P. pinaster* stem matrix, Table S2: Concentration range and calibration curves used for phytohormone quantification in 2-year-old *P. pinaster* stem tissues after pinewood nematode (PWN) inoculation.

## Appendix A

**Table A1:** Phytohormones (PH) quantification in stem tissues of 2-year-old *Pinus pinaster* half-sib plants (ng/g), 24, 48 and 72 h after inoculation (HAI) with the pinewood nematode (PWN) *Bursaphelenchus xylophilus*. Plant treatments include healthy non-inoculated (H), mock-inoculated/wounded (W), PWN-inoculated resistant (IN_R) and PWN-inoculated susceptible (IN_S). Data are presented as means ± SE of five independent biological replicates. Different letters indicate significant differences calculated using Tukey HSD test for multiple comparisons (*p* < 0.05).

PH	Treatment	ABA	GA	JA-ME	SA	ZeaR
24 HAI	H	162.9 ± 16.3 a	45.8 ± 7.4 a	615.7 ± 63.8 a	573.9 ± 125.5 a	0.153 ± 0.046 a
	W	198.4 ± 47.9 a	34.1 ± 1.0 a	577.3 ± 15.5 a	552.2 ± 151.0 a	0.086 ± 0.030 a
	IN_R	179.4 ± 73.3 a	34.1 ± 9.1 a	526.3 ± 106.6 a	526.3 ± 185.5 a	0.206 ± 0.043 a
	IN_S	159.1 ± 30.3 a	35.2 ± 8.4 a	703.8 ± 100.6 a	424.9 ± 96.2 a	0.120 ± 0.023 a
48 HAI	H	190.2 ± 40.7 ab	47.0 ± 6.7 a	578.0 ± 92.0 a	506.2 ± 138.6 b	0.144 ± 0.030 a
	W	207.1 ± 47.9 ab	42.0 ± 10.4 a	614.9 ± 86.3 a	421.0 ± 109.2 b	0.044 ± 0.008 a
	IN_R	113.9 ± 9.8 b	47.5 ± 12.6 a	455.7 ± 15.1 a	496.3 ± 210.5 b	0.109 ± 0.030 a
	IN_S	254.6 ± 25.2 a	47.7 ± 12.5 a	846.6 ± 91.7 a	1888.9 ± 657.9 a	0.136 ± 0.024 a
72 HAI	H	408.8 ± 164.7 a	48.8 ± 11.6 a	490.1 ± 68.6 b	681.9 ± 182.7 ab	0.100 ± 0.034 a
	W	134.0 ± 25.6 a	45.7 ± 9.5 a	561.5 ± 91.7 b	609.4 ± 155.9 ab	0.102 ± 0.019 a
	IN_R	209.3 ± 51.5 a	55.0 ± 16.4 a	655.1 ± 65.2 b	512.3 ± 246.6 b	0.167 ± 0.052 a
	IN_S	273.2 ± 39.7 a	36.6 ± 9.6 a	1131.5 ± 144.4 a	1583.4 ± 346.4 a	0.091 ± 0.019 a

## References

[1] Kuroda K., Zhao B.G., Futai K., Sutherland J.R., Takeuchi Y. (2008). Physiological incidences related to symptom development and wilting mechanism. Pine Wilt Disease.

[2] Mota M., Braasch H., Bravo M.A., Penas A.C., Burgermeister W., Metge K., Sousa E. (1999). First report of *Bursaphelenchus xylophilus* in Portugal and in Europe. Nematology.

[3] Rodrigues J.M., Mota M.M., Vieira P. (2008). National eradication programme for the pinewood nematode. Pine Wilt Disease: A Worldwide Threat to Forest Ecosystems.

[4] Sousa E., Rodrigues J.M., Bonifácio L.F., Naves P.M., Rodrigues A., Boeri F., Chung J.A. (2011). Management and control of the pine wood nematode, *Bursaphelenchus xylophilus*, in Portugal. Nematodes: Morphology, Functions and Management Strategies.

[5] EPPO (2018). PM 9/1 (6) *Bursaphelenchus xylophilus* and its vectors: Procedures for official control. EPPO Bull..

[6] Nunes da Silva M.N., Solla A., Sampedro L., Zas R., Vasconcelos M.W. (2015). Susceptibility to the pinewood nematode (PWN) of four pine species involved in potential range expansion across Europe. Tree Physiol..

[7] Zas R., Moreira X., Ramos M., Lima M.R.M., Nunes da Silva M., Solla A., Vasconcelos M.W., Sampedro L. (2015). Intraspecific variation of anatomical and chemical defensive traits in Maritime pine (*Pinus pinaster*) as factors in susceptibility to the pinewood nematode (*Bursaphelenchus xylophilus*). Trees.

[8] Rodrigues A.M., Mendes M.D., Lima A.S., Barbosa P.M., Ascensão L., Barroso J.G., Pedro L.G., Mota M.M., Figueiredo A.C. (2017). *Pinus halepensis*, *P. pinaster*, *P. pinea* and *P. sylvestris* essential oils chemotypes and monoterpene hydrocarbon enantiomers, before and after inoculation with the pinewood nematode *Bursaphelenchus xylophilus*. Chem. Biodiv..

[9] Carrasquinho I., Lisboa A., Inácio M.L., Gonçalves E. (2018). Genetic variation in susceptibility to pine wilt disease of maritime pine (*Pinus pinaster* Aiton) half-sib families. Ann. For. Sci..

[10] Menéndez-Gutiérrez M., Alonso M., Toval G., Díaz R. (2018). Testing of selected *Pinus pinaster* half-sib families for tolerance to pinewood nematode (*Bursaphelenchus xylophilus*). Forestry.

[11] Erb M., Kliebensteinb D.J. (2020). Plant secondary metabolites as defenses, regulators, and primary metabolites: The blurred functional trichotomy. Plant Physiol..

[12] Faria J.M.S., Sena I., Vieira da Silva I., Ribeiro B., Barbosa P., Ascensão L., Bennett R.N., Mota M., Figueiredo A.C. (2015). In vitro co-cultures of *Pinus pinaster* with *Bursaphelenchus xylophilus*: A biotechnological approach to study pine wilt disease. Planta.

[13] Kuroda H., Goto S., Kazumi E., Kuroda K. (2011). The expressed genes of Japanese red pine (*Pinus densiflora*) involved in the pine wilt disease severity. BMC Proc..

[14] Zhang F., Kajiwara J., Mori Y., Ohira M., Tsutsumi Y., Kondo R. (2013). Metabolites from resistant and susceptible *Pinus thunbergii* after inoculation with pine wood nematode. Am. J. Plant Sci..

[15] Pimentel C.S., Gonçalves E.V., Firmino P.N., Calvão T., Fonseca L., Abrantes I., Correia O., Máguas C. (2017). Differences in pine species constitutive and inducible defences determining the susceptibility to the pinewood nematode. Plant Pathol..

[16] Canas S., Trindade C.S., Sun B., Naves P. (2020). Phenolic compounds involved in pine wilt disease: HPLC-based method development and validation for their quantification. J. Plant Biochem. Biotechnol..

[17] Gaspar D., Trindade C., Usié A., Meireles B., Barbosa P., Fortes A.M., Pesquita C., Costa R.L., Ramos A.M. (2017). Expression profiling in *Pinus pinaster* in response to infection with the pine wood nematode *Bursaphelenchus xylophilus*. Forests.

[18] Liu Q., Wei Y., Xu L., Hao Y., Chen X., Zhou Z. (2017). Transcriptomic profiling reveals differentially expressed genes associated with pine wood nematode resistance in Masson Pine (*Pinus massoniana* Lamb.). Sci. Rep..

[19] Lee I.H., Kim J., Woo K.S., Jang K.-H., Kim Y.-H., Shim D. (2018). De novo assembly and transcriptome analysis of the *Pinus densiflora* response to pine wilt disease in nature. Plant Biotechnol. Rep..

[20] Lee I.H., Han H., Koh Y.H., Kim I.S., Lee S.-W., Shim D. (2019). Comparative transcriptome analysis of *Pinus densiflora* following inoculation with pathogenic (*Bursaphelenchus xylophilus*) or non-pathogenic nematodes (*B. thailandae*). Sci. Rep..

[21] Bari R., Jones J.D.G. (2009). Role of plant hormones in plant defence responses. Plant Mol. Biol..

[22] Wina S.H., Kumar V., Shriram V., Sah S.K. (2016). Phytohormones and their metabolic engineering for abiotic stress tolerance in crop plants. Crop J..

[23] Dubois M., Van den Broeck L., Inzé D. (2018). The pivotal role of ethylene in plant growth. Trends Plant Sci..

[24] Robert-Seilaniantz A., Grant M., Jones J.D.G. (2011). Hormone crosstalk in plant disease and defense: More than just jasmonates-salicylate antagonism. Annu. Rev. Phytopathol..

[25] Pieterse C., van der Does D., Zamioudis C., Leon-Reyes A., van Wees S.C. (2012). Hormonal modulation of plant immunity. Annu. Rev. Cell Dev. Biol..

[26] Thaler J.S., Humphrey P.T., Whiteman N.K. (2012). Evolution of jasmonate and salicylate signal crosstalk. Trends Plant Sci..

[27] Verma V., Ravindran P., Kumar P.P. (2016). Plant hormone-mediated regulation of stress responses. BMC Plant Biol..

[28] Davies P.J., Davies P.J. (2010). The plant hormones: Their nature, occurrence, and functions. Plant Hormones.

[29] Fu J., Sun X., Wang J., Chu J., Yan C. (2011). Progress in quantitative analysis of plant hormones. Sci. Bull..

[30] Rawlinson C., Kamphuis L.G., Gummer J.P.A., Singh K.B., Trengove R.D. (2015). A rapid method for profiling of volatile and semi-volatile phytohormones using methyl chloroformate derivatization and GC-MS. Metabolomics.

[31] Seo H., Kriechbaumer V., Park W.J. (2016). Modern quantitative analytical tools and biosensors for functional studies of auxin. J. Plant Biol..

[32] Cao D., Lutz A., Hill C.B., Callahan D., Roessner U. (2017). A quantitative profiling method of phytohormones and other metabolites applied to barley roots subjected to salinity stress. Front. Plant Sci..

[33] Pan X., Welti R., Wang X. (2008). Simultaneous quantification of major phytohormones and related compounds in crude plant extracts by liquid chromatography–electrospray tandem mass spectrometry. Phytochemistry.

[34] Pan X., Wang X. (2009). Profiling of plant hormones by mass spectrometry. J. Chromatogr. B.

[35] Trapp M.A., de Souza G.D., Rodrigues-Filho E., Boland W., Mithӧfer A. (2014). Validated method for phytohormone quantification in plants. Front. Plant Sci..

[36] Ross A.R.S., Ambrose S.J., Cutler A.J., Feurtado A., Kermose A.R., Nelson K., Zhou R., Abrams S.R. (2004). Determination of endogenous and supplied deuterated abscisic acid in plant tissues by high-performance liquid chromatography-electrospray ionization tandem mass spectrometry with multiple reaction monitoring. Anal. Biochem..

[37] Wang S., Chen L., Fan C.Q., Wang P. (2016). Determination of abscisic acid, gibberellic acid, indole-3-acetic acid, and zeatin riboside in masson pine [*Pinus massoniana* L.] by accelerated solvent extraction and high-performance liquid chromatography—Tandem mass spectrometry. Anal. Lett..

[38] Delatorre C., Rodríguez A., Rodríguez L., Majada J.P., Ordás R.J., Feito I. (2017). Hormonal profiling: Development of a simple method to extract and quantify phytohormones in complex matrices by UHPLC–MS/MS. J. Chromatogr. B.

[39] Rodrigues A.M., António C., António C. (2018). Standard key steps in mass spectrometry-based plant metabolomics experiments: Instrument performance and analytical method validation. Plant Metabolomics. Methods in Molecular Biology.

[40] Moosavi S.M., Ghassabian S. (2018). Linearity of calibration curves for analytical methods: A review of criteria for assessment of method reliability. Calibration and Validation of Analytical Methods—A Sampling of Current Approaches.

[41] Pan X., Welti R., Wang X. (2010). Quantitative analysis of major plant hormones in crude plant extracts by high-performance liquid chromatography-mass spectrometry. Nat. Protoc..

[42] Balcke G.U., Handrick V., Bergau N., Fichtner M., Henning A., Stellmach H., Tissier A., Hause B., Frolov A. (2012). An UPLC-MS/MS method for highly sensitive high-throughput analysis of phytohormones in plant tissues. Plant Methods.

[43] Ma L., Zhang H., Xu W., He X., Yang L., Luo Y., Huang K. (2013). Simultaneous determination of 15 plant growth regulators in bean sprout and tomato with liquid chromatography-triple quadrupole tandem mass spectrometry. Food Anal. Methods.

[44] Floková K., Tarkowská D., Miersch O., Strnad M., Wasternack C., Novák O. (2014). UHPLC–MS/MS based target profiling of stress-induced phytohormones. Phytochemistry.

[45] Wu Z., Gao W., Phelps M.A., Wu D., Miller D.D., Dalton J.T. (2004). Favorable effects of weak acids on negative-ion electrospray ionization mass spectrometry. Anal. Chem..

[46] Hua Y., Jenke D. (2012). Increasing the sensitivity of an LC-MS method for screening material extracts for organic extractables via mobile phase optimization. J. Chromatogr. Sci..

[47] Kowalczyk M., Sandberg G. (2001). Quantitative analysis of indole-3-acetic acid metabolites in Arabidopsis. Plant Physiol..

[48] Correia B., Pintó-Marijuan M., Castro B.B., Brossa R., López-Carbonell M., Pinto G. (2014). Hormonal dynamics during recovery from drought in two *Eucalyptus globulus* genotypes: From root to leaf. Plant Physiol. Biochem..

[49] Thakare R., Chhonker Y.S., Gautam N., Alamoudi J.A. (2016). Quantitative analysis of endogenous compounds. J. Pharm. Biomed. Anal..

[50] Koek M.M., Jellema R.H., van der Greef J., Tas A.C., Hankemeier T. (2011). Quantitative metabolomics based on gas chromatography mass spectrometry: Status and perspectives. Metabolomics.

[51] George E.F., Hall M.A., Klerk G.J.D., George E.F., Hall M.A., Klerk G.J.D. (2008). Plant growth regulators I: Introduction; auxins, their analogues and inhibitors. Plant Propagation by Tissue Culture.

[52] Hirao T., Fukatsu E., Watanabe A. (2012). Characterization of resistance to pine wood nematode infection in *Pinus thunbergii* using suppression subtractive hybridization. BMC Plant Biol..

[53] Balint-Kurti P. (2019). The plant hypersensitive response: Concepts, control and consequences. Mol. Plant Pathol..

[54] Myers R.F. (1988). Pathogenesis in pine wilt caused by pinewood nematode, *Bursaphelenchus xylophilus*. J. Nematol..

[55] Van Loon L.C., Rep M., Pieterse C.M.J. (2006). Significance of inducible defense related proteins in infected plants. Annu. Rev. Phytopathol..

[56] Evans S., Evans H., Ikegami M., Mota M.M., Vieira P. (2008). Modeling PWN-induced wilt expression: A mechanistic approach. Pine Wilt Disease: A Worldwide Threat to Forest Ecosystems.

[57] Li Z., Zhang Q., Zhou X. (2016). A 2-Cys peroxiredoxin in response to oxidative stress in the pine wood nematode, *Bursaphelenchus xylophilus*. Sci. Rep..

[58] Yin R., Liu X., Yu J., Ji Y., Liu J., Cheng L., Zhou J. (2020). Up-regulation of autophagy by low concentration of salicylic acid delays methyl jasmonate-induced leaf senescence. Sci. Rep..

[59] Cardoso A.A., Gori A., Da-Silva C.J., Brunetti C. (2020). Abscisic acid biosynthesis and signaling in plants: Key targets to improve water use efficiency and drought tolerance. Appl. Sci..

[60] Denancé N., Sánchez-Vallet A., Goffner D., Molina A. (2013). Disease resistance or growth: The role of plant hormones in balancing immune responses and fitness costs. Front. Plant Sci..

[61] Mauch-Mani B., Mauch F. (2005). The role of abscisic acid in plant-pathogen interactions. Curr. Opin. Plant Biol..

[62] Ribeiro B., Espada M., Vu T., Nóbrega F., Mota M., Carrasquinho I. (2012). Pine wilt disease: Detection of the pinewood nematode (*Bursaphelenchus xylophilus*) as a tool for a pine breeding programme. For. Pathol..

[63] Matuszewski B.K., Constanzer M.L., Chavez-Eng C.M. (2003). Strategies for the assessment of matrix effect in quantitative bioanalytical methods based on HPLC-MS/MS. Anal. Chem..

[64] Hartmann C., Smeyers-Verbeke J., Massart D.L., McDowall R.D. (1998). Validation of bioanalytical chromatographic methods. J. Pharm. Biomed. Anal..

[65] RStudio Team (2018). RStudio: Integrated Development for R.

[66] R Core Team (2019). R: A Language and Environment for Statistical Computing.

[67] Mendiburu F. (2020). Agricolae: Statistical Procedures for Agricultural Research. R Package Version 1.3-3. https://CRAN.R-project.org/package=agricolae.

[68] Warnes G.R., Bolker B., Bonebakker L., Gentleman R., Huber W., Liaw A., Thomas L., Maechler M., Magnusson A., Moeller S. (2020). Gplots: Various R Programming Tools for Plotting Data. R Package Version 3.0.3. https://CRAN.R-project.org/package=gplots.

