# Supplementary material for: Pinus pinaster Early Hormonal Defence Responses to Pinewood Nematode (Bursaphelenchus xylophilus) Infection
Source: Metabolites. 2021 Apr 8;11(4):227. doi: 10.3390/metabo11040227 (PMC8068127; doi:10.3390/metabo11040227)
Supplement: Supplementary file 1 [file metabolites-11-00227-s001.pdf]

metabolites

MDPI

Supplementary material

Pinus pinaster early hormonal defence responses to pinewood nematode (*Bursaphelenchus xylophilus*) infection

Table S1. Phytohormones (PH) and stable isotope-labeled internal standards (IS) used in this work.

Analyte	Abbreviation	Class	Supplier	Product Number
(±)-9,10-Dihydrojasmonic acid	DHJA	IS	OlChemIm s.r.o.	0145321
(±)-9,10-Dihydrojasmonic acid methyl ester	DHJA-ME	IS	OlChemIm s.r.o.	0145671
[$^2H_2$] Gibberellin A9	d_2-GA9	IS	OlChemIm s.r.o.	0322661
[$^2H_4$] Salicylic acid	d_4-SA	IS	OlChemIm s.r.o.	0376581
[$^2H_5$] Benzoic acid	d_5-BA	IS	OlChemIm s.r.o.	0376591
[$^2H_5$] Indole-3-acetic acid	d_5-IAA	IS	OlChemIm s.r.o.	0311531
[$^2H_5$] Indole-3-acetic acid methyl ester	d_5-IAA-ME	IS	OlChemIm s.r.o.	0311551
[$^2H_5$] *trans*-Zeatin	d_5-Zea	IS	OlChemIm s.r.o.	0300301
[$^2H_6$] Abscisic acid	d_6-ABA	IS	OlChemIm s.r.o.	0034221
Abscisic acid	ABA	PH	Sigma-Aldrich	A1049
Benzoic acid	BA	PH	Sigma-Aldrich	242381
Gibberellic acid	GA	PH	Sigma-Aldrich	36575
Gibberellin A_9	GA_9	PH	OlChemIm s.r.o.	0122661
Indole-3-acetic acid	IAA	PH	Sigma-Aldrich	45533
Indole-3-acetic acid methyl ester	IAA-ME	PH	OlChemIm s.r.o.	0031551
Indole-3-butyric acid	IBA	PH	OlChemIm s.r.o.	0031661
Indole-3-butyric acid methyl ester	IBA-ME	PH	OlChemIm s.r.o.	0035431
Jasmonic acid	JA	PH	Sigma-Aldrich	14631
Jasmonic acid methyl ester	JA-ME	PH	Sigma-Aldrich	392707
N_6-Isopentenyladenine	iP	PH	OlChemIm s.r.o.	0010161
Salicylic acid	SA	PH	Sigma-Aldrich	247588
trans-Zeatin	Zea	PH	Sigma-Aldrich	Z0164
trans-Zeatin riboside	ZeaR	PH	OlChemIm s.r.o.	0010311

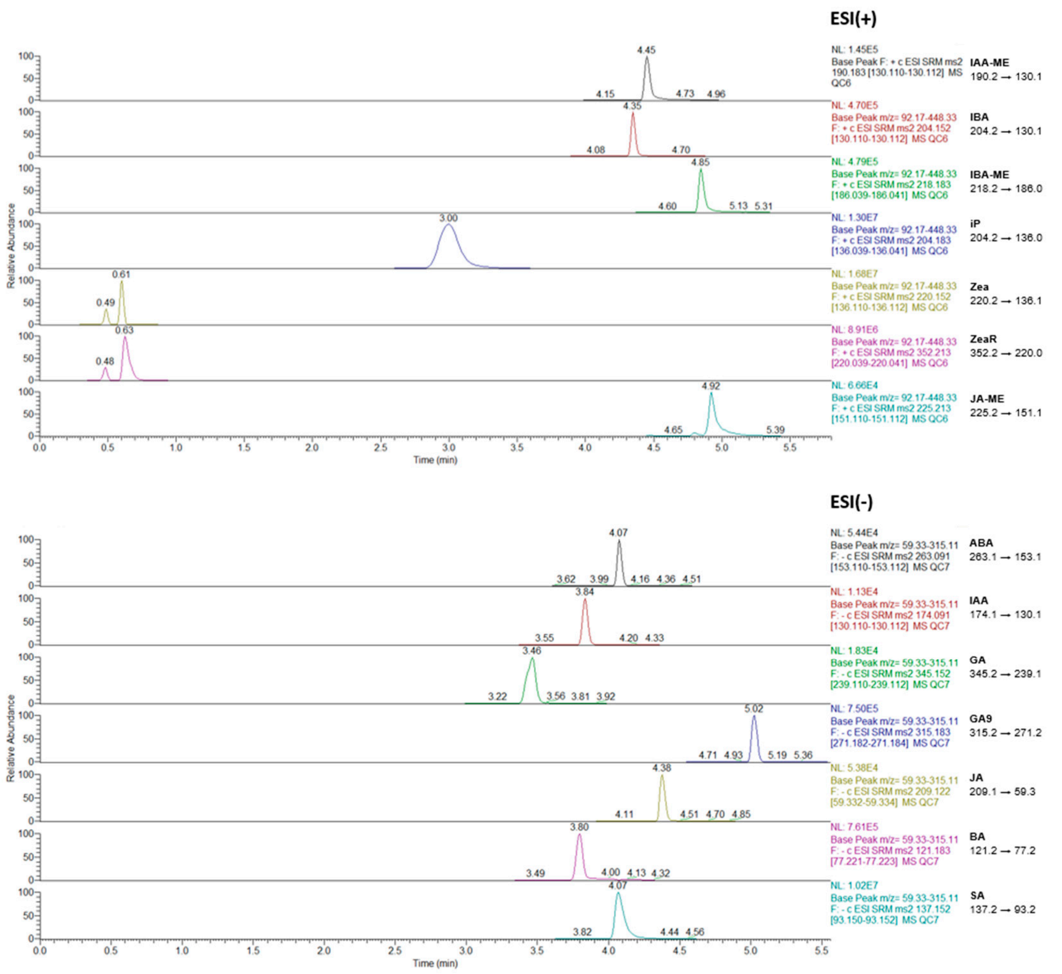

Figure S1. Selected reaction monitoring (SRM) chromatograms obtained using ESI+ and ESI- for the LC-QqQ-MS/MS separation of 14 target phytohormones (1 μg/mL). For SRM parameters see Table 1 in the main text.

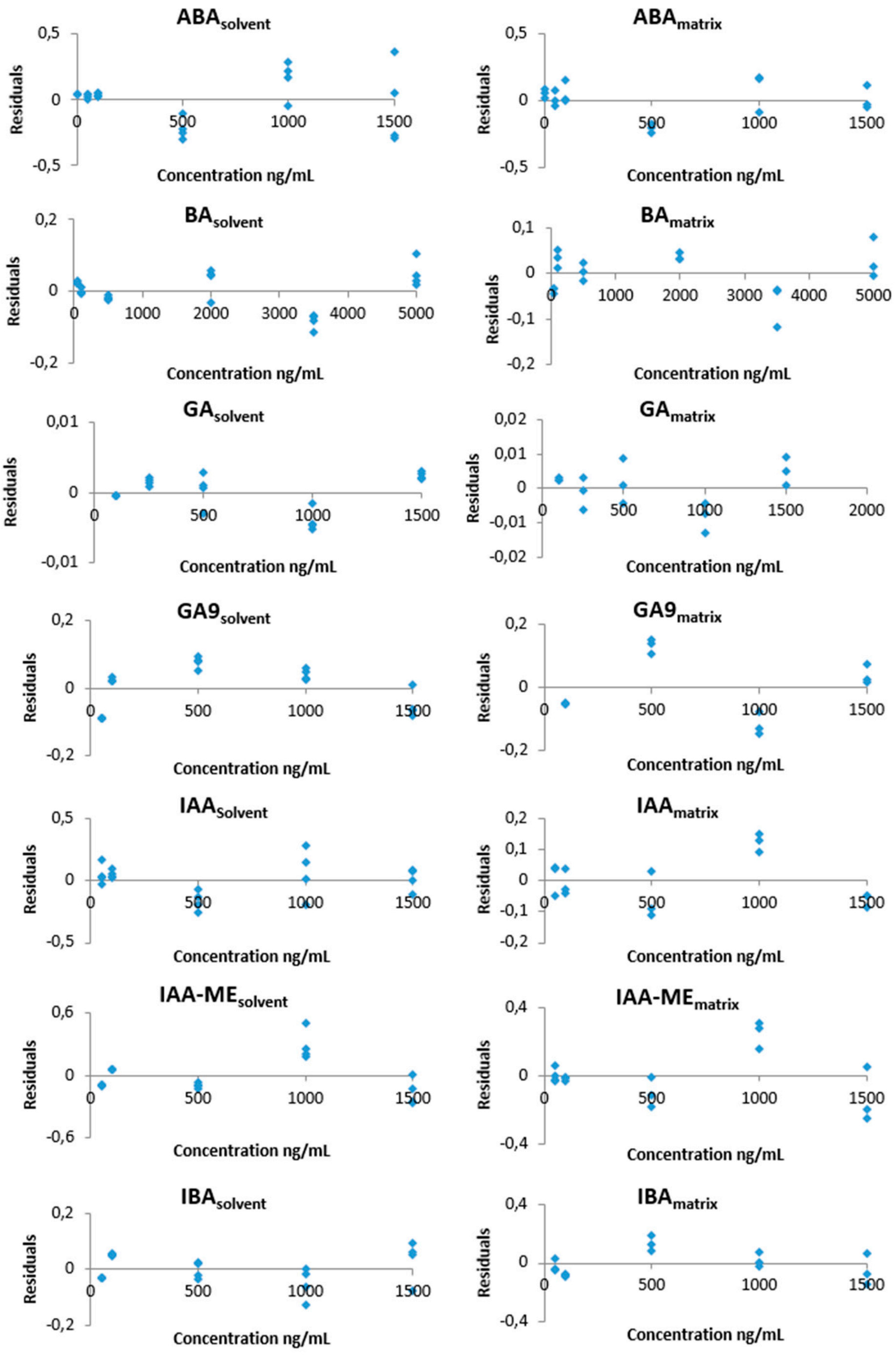

Figure S2. Residual plots for evaluating the homoscedasticity and goodness of fit of the calibration curves prepared in solvent and in 1-year old *P. pinaster* stem matrix, for abscisic acid (ABA, at 1500, 1000, 500, 100, 50, 5 ng/mL), benzoic acid (BA, at 5000, 3500, 2000, 500, 100, 50), gibberellic acid (GA, at 1500, 1000, 500, 250, 100 ng/mL), gibberellin A9 (GA_9, at 1500, 1000, 500, 100, 50), indole-3-acetic acid (IAA, at 1500, 1000, 500, 100, 50 ng/mL), indole-3-acetic acid methyl ester (IAA-ME, at 1500, 1000, 500, 100, 50 ng/mL), indole-3-butyric acid (IBA at 1500, 1000, 500, 100, 50 ng/mL).

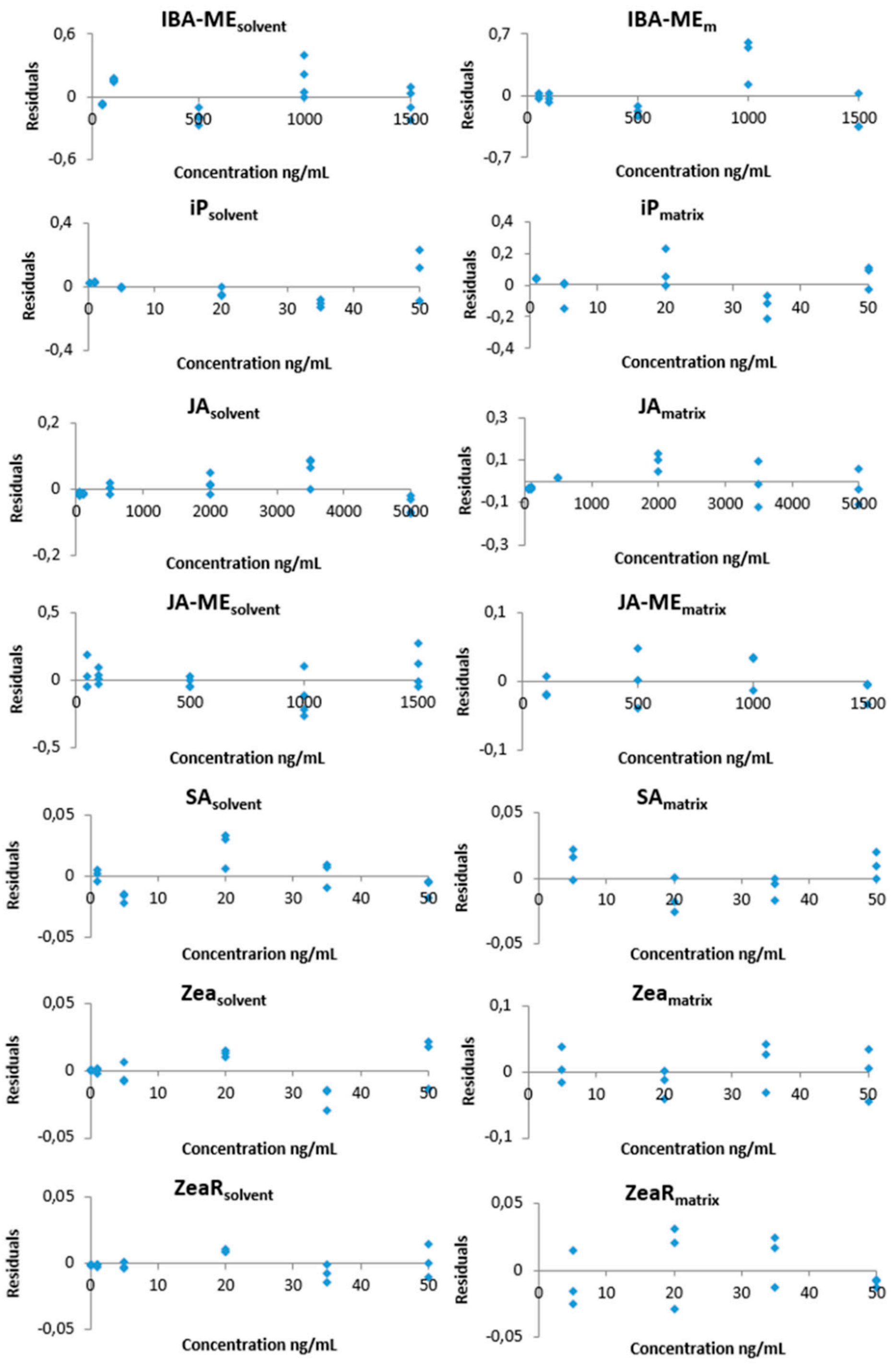

Figure S3. Residual plots for evaluating the homoscedasticity and goodness of fit of the calibration curves prepared in solvent and in 1-year old *P. pinaster* stem matrix, for indole-3-butyric acid methyl ester (IBA-ME at 1500, 1000, 500, 100, 50 ng/mL), N_6-isopentenyladenine (iP, at 50, 35, 20, 5, 1, 0.1 ng/mL), jasmonic acid (JA, at 5000, 3500, 2000, 500, 100, 50 ng/mL), jasmonic acid methyl ester (JA-ME, at 1500, 1000, 500, 100, 50 ng/mL), salicylic acid (SA, at 50, 35, 20, 5, 1 ng/mL), *trans*-zeatin (Zea, at 50, 35, 20, 5, 1, 0.1 ng/mL), *trans*-zeatin riboside (ZeaR at 50, 35, 20, 5, 1, 0.1 ng/mL).

Table S2. Concentration range and calibration curves used for phytohormone quantification in 2-year-old *Pinus pinaster* stem tissues after pinewood nematode (PWN) inoculation.

**Analyte**	**Concentration range (ng/mL)**	**Calibration curve**
ABA	500, 250, 125, 25, 5, 1	0.0050835x – 0.019533, R^2 = 0.98
BA	2000, 1000, 500, 100, 20, 4	0.0013109x + 0.01277, R^2 = 0.99
GA	10000, 5000, 2500, 500, 100, 20	2.9796x + 5.8663, R^2 = 0.99
GA_9	5000, 2500, 1250, 250, 50, 10	82,628x + 832,51, R^2 = 0.98
IAA	3000, 1500, 750, 150, 30, 6	0.00026221x + 0.013823, R^2 = 0.97
IAA-ME	5000, 2500, 1250, 250, 50, 10	0.0056105x + 0.16635, R^2 = 0.98
IBA	2000, 1000, 500, 100, 20, 4	0.023678x – 0.14465, R^2 = 0.99
IBA-ME	5000, 2500, 1250, 250, 50, 10	0.023209x + 0.089531, R^2 = 0.99
JA	500, 250, 125, 25, 5, 1	0.019902x + 0.064494, R^2 = 0.99
JA-ME	5000, 2500, 1250, 250, 50, 10	0.18833x + 20.755, R^2 = 0.96
SA	5000, 2500, 1250, 250, 50, 10	0.0022136x + 0.013222, R^2 = 0.99
Zea	100, 50, 25, 5, 1, 0.2	0.0077849x + 0.0010627, R^2 = 0.99
ZeaR	50, 25, 12.5, 2.5, 0.5, 0.1	0.057090x + 0.0032366, R^2 = 0.99
